# Detection of Alzheimer’s disease (AD) specific tau pathology with conformation-selective anti-tau monoclonal antibody in co-morbid frontotemporal lobar degeneration-tau (FTLD-tau)

**DOI:** 10.1186/s40478-019-0687-5

**Published:** 2019-03-04

**Authors:** Garrett S. Gibbons, Soo-Jung Kim, John L. Robinson, Lakshmi Changolkar, David J. Irwin, Leslie M. Shaw, Virginia M.-Y. Lee, John Q. Trojanowski

**Affiliations:** 1 Department of Pathology and Laboratory Medicine, Institute on Aging and Center for Neurodegenerative Disease Research, 3600 Spruce St. 3 Maloney, Philadelphia, PA 19104 USA; 2 Department of Neurology, University of Pennsylvania School of Medicine, Philadelphia, PA 19104 USA

**Keywords:** Alzheimer’s disease, Frontotemporal lobar degeneration, Tau, Tauopathy, Monoclonal antibody

## Abstract

Pathological tau aggregates in Alzheimer’s disease (AD) and frontotemporal lobar degeneration-tau (FTLD-tau) adopt distinct conformations differentiated by the AD-tau specific monoclonal antibody (mAb) GT-38 that are not readily visualized using phosphorylation-specific anti-tau mAbs. To determine the extent of co-morbid AD-tau pathology in FTLD-tau, we performed immunohistochemical (IHC) staining with GT-38 and assigned Braak stages of AD-tau in a cohort 180 FTLD-tau cases consisting of corticobasal degeneration (CBD; *n* = 49), progressive supranuclear palsy (PSP; *n* = 109), and Pick’s disease (PiD; *n* = 22). Nearly two-thirds of patients (*n* = 115 of 180, 63.8%) with FTLD-tau had some degree of comorbid AD-tau pathology and 20.5% of the FTLD-tau cohort had Braak stage ≥B2, consistent with medium-to-high-level AD neuropathological change (ADNPC). The PSP group had the highest frequency of medium-high AD-tau pathology compared to other tauopathies (PSP = 31/109, 28.4%; Picks = 2/22, 9.1%, CBD = 4/49, 8.2%) but neuropathological diagnosis was not found to be a significant independent predictor of medium-high AD Braak stage in a multivariate model after accounting for age at death (OR = 1.09; 95% CI = 1.03–1.15; *p* = 0.002) and CERAD plaque scores (OR = 3.75, 95% CI = 1.58–8.89; *p* = 0.003), suggesting there is no predilection for a specific FTLD tauopathy to develop AD-tau co-pathology after accounting for age. Patients with FTLD-tau who had, clinically significant, medium-high AD-tau pathology had significantly higher antemortem CSF levels of both total-tau (t-tau; mean = 89.98 pg/ml, SD = 36.70 pg/ml) and phosphorylated-tau (p-tau; mean = 20.45 pg/ml, SD = 9.31 pg/ml) compared to patients with negligible-low AD-tau, t-tau (mean = 43.04 pg/ml, SD = 25.40 pg/ml) and p-tau (mean = 11.90 pg/ml, SD = 4.48 pg/ml) (*p* ≤ 0.001 both). Finally, in an exploratory analysis in our largest pathology group (PSP) we find an association of GT-38 AD-tau Braak stage with lower baseline MMSE (*p* = 0.03). Together, these finding validate the use of GT-38 to selectively detect AD-tau pathology in the context of FTLD-tau and provides a novel tool to investigate associations of clinical phenotypes amongst co-morbid tauopathies.

## Introduction

Pathological aggregates of tau protein within the central nervous system are a neuropathological hallmark of a heterogeneous class of diseases termed tauopathies [39]. The normal function of tau is to stabilize microtubules within axons in the central nervous system, mediated by microtubule binding domain (MTBRs) [11, 24]. Tau is natively unstructured and expressed as 6 isoforms in the adult human brain as the result of alternative splicing and contains either 0-2 N-terminal acidic domains (0-2N) and 3 or 4 MTBRs (3R or 4R) [20]. Although primarily expressed in neurons, there is evidence of low levels of tau expression in glial cells [4, 41, 56]. Mutations of the tau encoding *MAPT* gene on chromosome 17 are responsible for familial frontotemporal lobar degeneration-tau (FTLD-tau); formerly referred to as frontotemporal degeneration and parkinsonism linked to chromosome 17 (FTDP-17) [16, 26, 27]. Under pathological conditions, imparted by *MAPT* mutations or sporadically, tau adopts a beta-sheet structure and forms amyloid-fibrils within neurons and glia [19, 57].

Alzheimer’s disease (AD) and FTLD-tau including corticobasal degeneration (CBD), progressive supranuclear palsy (PSP) and Pick’s disease (PiD) are composed of morphologically and cell-type-specific pathological tau aggregates [39]. Growing evidence supports the notion that distinct tauopathies are composed of “strains” of tau that represent structural polymorphisms, or conformations, that can be stably passaged in cell culture and *in vivo*. For instance, insoluble paired helical filaments (PHF) of tau derived from post-mortem tissue of humans with AD (AD-tau) are internalized by wildtype murine primary neurons and recruit endogenous mouse tau, propagating insoluble fibrils [23]. On intracerebral injections of insoluble tau derived from humans with CBD or PSP into wildtype mice, endogenous mouse tau is recruited into tau aggregates that recapitulate the cell-type specificity and morphology of their human tauopathy counterparts [44]. Furthermore, tau aggregates derived from different tauopathies have unique trypsin-resistant fragments characterized by mass-spectrometry suggesting tau adopts distinct conformations with varying accessibility to trypsin digestion sites [53]. Finally, pathological tau strains may be defined by isoform composition in addition to structural conformations since tau aggregates in CBD and PSP are composed primarily of 4R tau isoforms, whereas PiD aggregates are composed primarily of 3R tau isoforms [29].

Post-mortem neuropathological analysis of tau by immunohistochemistry is performed using standard diagnostic antibodies that detect phosphorylated tau (AT8 or PHF1) or the pathological conformation of tau aggregates (MC1) [6, 37]. The stereotypical spread of tau pathology from the locus coeruleus and trans entorhinal cortex to the hippocampus and eventually multiple cortical regions allows for the designation of 6 stages of pathological tau in AD (Braak stages I-VI) [7]. Since both AD-tau and FTLD-tau share many post-translational modifications, previously available tau antibodies failed to distinguish between these forms of tauopathy confounding the assessment of Braak AD-tau staging in patients with co-existent FTLD-tau [30, 31, 36, 47]. This limitation has been addressed partially by utilizing the amyloid-binding dye Thioflavin S (Thio S), in which AD tauopathy has robust reactivity to amyloid-binding dyes while FTLD-tau does not [33]. However, this approach has limitations since Thio S is an amyloid binding dye that is not specific for tau and also detects amyloid beta and rare subsets of TDP-43, as well as alpha-synuclein aggregates [32]. Thus, a marker specific to AD-tau has the potential to improve neuropathological diagnoses and could potentially be extended into *in vivo* studies to differentiate tauopathies during life. Therefore, we developed anti-tau conformation-selective mAbs, GT-7 and GT-38, that selectively detect AD-tau but not CBD, PSP, or PiD by immunizing mice with human brain derived AD-tau enriched extracts comprised of both 3R and 4R tau [18]. To assess the prevalence of AD-tau co-pathology in FTLD-tau we performed immunohistochemical analysis with GT-38 to determine for the first time the AD-tau Braak stage in a cohort of 180 patients with neuropathologically confirmed FTLD-tau. We then extended these findings to a subset of the same patients from whom we obtained CSF during life that were analyzed for levels of tau and Aβ.

## Materials and methods

### Patients

Cases of FTLD-tau were selected from the University of Pennsylvania Center for Neurodegenerative Disease Research (CNDR) brain bank following neuropathological diagnostic evaluation according to current neuropathological criteria as described [1, 15, 28, 29, 42, 55]. Patient demographics, post-mortem interval, a pathological diagnosis were obtained from the Integrated Neurodegenerative Disease Database (INDD) at CNDR [59]. Clinical diagnoses and baseline Folstein mini-mental state examination (MMSE) scores were extracted from INDD in our largest pathology group (PSP) for exploratory clinicopathological correlations.

### Generating GT-38 IgG2a

GT-38 IgG1 heavy and light chain variable fragments were sequenced from monoclonal hybridoma cell populations (Lake Pharma, Belmont, CA). A gene encoding the GT-38 IgG2a heavy chain was designed to incorporate the GT-38 heavy chain variable domain and inserted into the mouse IgG2a chain C region A allele of the *Ighg* gene (Uniprot ID P01863) and the variable light chain was adjoined to the immunoglobulin kappa constant region *Igkc* gene (Uniprot ID P01837). The oligonucleotide sequences were synthesized and cloned into pCDA3(-) vectors (GenScript, Piscataway, NJ). QBI293 cells were transiently co-transduced with the GT-38 IgG2a heavy chain and kappa light chain vectors with Lipofectamine3000, 10 μg cDNA per 10 cm dish. Cells were cultured in DMEM with 10% fetal bovine serum cleared of IgG, penicillin, and streptomycin. Media was collected and replaced after 72 hr co-transfection and GT-38 IgG2a purified by mAb SelectSure 5 mL column (GE Healthcare), eluted with 100 mM glycine pH 3.0, 150 mM sodium chloride and neutralized in 1 M Tris pH 9.0, then buffer exchanged into phosphate buffered saline (PBS).

### Co-immunofluorescent (co-IFC) and immunohistochemical (IHC) staining

Tissue obtained at autopsy was fixed in 70% ethanol and 150 mM sodium chloride or 10% neutral buffered formalin, paraffin embedded, and cut into 6 μm thick sections. Tissue was deparaffinized in xylene and rehydrated in ethanol (100%-70%) and treated with 7.5% hydrogen peroxide in methanol for 30 min at room temperature then washed in water 10 min as described for IHC [18]. Antigen retrieval was performed with citric acid unmasking solution (H-3300, Vector Laboratories) heated at 95°C for 15 min and allowed to slowly cool to room temperature. Tissue was blocked with 2% FBS in 100 mM Tris pH 7.2 for 5 min at room temperature. Co-IFC was performed using IgG2a GT-38 at a final concentration of 2 μg/mL and mouse IgG1 isotype antibodies specific to 3R tau, RD3 (05-803, Millipore), and 4R tau, RD4 (05-804, Millipore), diluted 1:1,000 in 2% FBS 0.1 M Tris pH 7.2 at 4°C overnight. Fluorescent conjugated secondary antibodies, anti-mouse IgG2a 488 and anti-mouse IgG1 594 were diluted 1:1,000 in 2% FBS 0.1 M Tris pH 7.2 and incubated for 2 hr at room temperature. Tissue was washed twice for 5 min in 0.1 M Trish pH 7.2 and autofluorescence quenched by 20 sec incubation in Sudan Black solution followed by 5 sec rinse in 70% ethanol and 10 min wash in water. Slides were coverslipped with Fluoromount containing DAPI (0100-20, Southern Biotech).

For IHC, GT-38 IgG1 was used at a final concentration of 2 μg/mL and PHF1 IgG1 (gift of Peter Davies) was diluted 1:1,000 in in 2% FBS 0.1 M Tris pH 7.2 and incubated at 4°C overnight. Binding was detected with biotinylated anti-mouse secondary antibody (BA-2000, Vector Laboratories) and developed with Vectastain ABC kit (PK-6100, Vector Laboratories) and ImmPACT DAB (SK-4105 Vector Laboratories) for 5 min. Tissue was dehydrated in a series of ethanol (70%-100%) and xylene, then coverslipped with Cytoseal 60 (8310-4, Thermo).

### Braak staging AD-tau

Braak staging was performed using standard criteria by evaluation of hippocampus (including the cornu amonis subfields, enterorhinal cortex and adjacent fusiform temporal neocortex) and visual cortex sections by IHC stained with GT-38 in accordance with standard diagnostic criteria [6, 42]. We also included a brainstem region in the upper pons to capture an area vulnerable to FTLD-tau and relatively spared in AD [28, 31]. Each case was assigned a Braak stage B0-B3 based on the distribution of GT-38 positive tau pathology. Ordinal scores from 0-3+ were assigned for GT-38 staining intensity in the transentorhinal cortex, CA regions, dentate gyrus, basis pontis, and visual cortex.

### Cerebrospinal fluid (CSF) analysis

CSF was collected from living patients in accordance with standard operating procedures as previously described [34]. Total tau (t-tau), phosphorylated (p-tau) tau, and amyloid-β 1-42 (Aβ_1-42_) were determined using either the Innotest enzyme linked immunosorbent assay (ELISA); Fujirebio-Europe or INNO-BIA AlzBio 3x MAP Luminex platforms. Absolute values from different platforms were transformed into equivalent xMAP units for comparison using previously reported validated algorithms [14, 52, 58].

### Statistical analysis

Patient demographic variables were assessed for normal distribution with Shapiro-Wilk normality test. Differences in demographics between FLTD-tau subtypes were tested by Kruskal-Wallis test for non-normally distributed data with Dunn’s multiple comparison post-hoc test and ANOVA with Bonferroni’s multiple comparisons test for normally distributed data. Chi-squared test was used to assess the distribution of Braak stages among categorical diagnostic categories of FTLD-tau subtypes (i.e. PSP, CBD, PiD) and Consortium to Establish a Registry for Alzheimer’s Disease (CERAD) plaque score stages. We used the collapsed Braak I-VI stages where I-II are referred to as B1, III-IV as B2, and V-VI as B3. Based on standard neuropathological criteria and the low number of individual patients with AD Braak stages B2 and B3, we compared Braak stages B0/B1 with negligible-low AD-tau compared to Braak stages B2/B3 with medium-high levels of AD-tau pathology sufficient to contribute to clinical dementia [42]. Moreover, we collapsed categorical CERAD stages into C0/C1 and C2/C3 based on the small sample size of patients with high CERAD scores. Multivariate regression models were used to test the association of the outcome of medium-high level AD (B2/B3) vs. negligible-low AD-tau (B0/B1) co-pathology as the dependent variable of logistic regressions with either age at death, age at onset, FTLD-tau subtype (PSP, CBD, PiD), sex and CERAD score (C0/1 vs. C2/3) as independent predictors. We used Bayesian information criteria (BIC) to guide model selection for the optimal multivariate model reported. Mann-Whitney rank sum test was used to assess differences of total tau, phosphorylated tau, and Aβ_1-42_ CSF levels between negligible-low AD-tau and medium-high AD-tau groups. For clinicopathological correlations of baseline MMSE in PSP we collapsed Braak B3 group (*n*=1) with Braak B2 for Kruskal-Wallis analysis across B0, B1, B2/3 groups with planned post-hoc Mann-Whitney U analyses between individual groups.

## Results

### Detection of comorbid AD-tau pathology in FTLD-tau

**Fig. 1:**
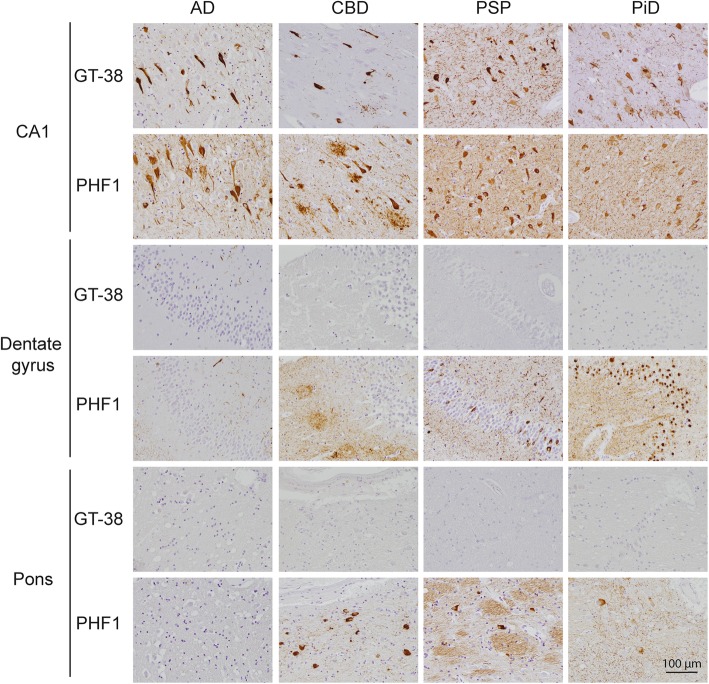
GT-38 selectively detects AD-tau pathology in the context of co-occurring FTLD-tau. IHC staining of hippocampus CA1, dentate gyrus, and pons with GT-38 or phospho-tau antibody PHF1 demonstrates that GT-38 detects tau NFTs in CA1 region but not neuronal inclusions in the molecular layer of the dentate gyrus present in FTLD-tau. GT-38 does not detect neuronal or astrocytic tau pathology in pons of FTLD-tau cases verifying selectivity of GT-38 for AD-tau pathology

We recently reported the AD-tau specific mAb GT-38 selectively detects AD-tau pathology but not tau pathology diagnostic of CBD, PSP, or PiD [18]. We expanded on these findings in a cohort of patients with neuropathologically diagnosed FTLD-tau to evaluate the selectivity of GT-38 to detect AD-tau pathology in the context of comorbid FTLD-tau. GT-38 selectively detected typical AD-tau morphologies including neurofibrillary tangles (NFTs), neuritic plaques, and neuropil thread tau pathology in the entorhinal cortex and CA regions of the hippocampus in cases of FTLD-tau (Fig. 1). The phospho-tau diagnostic antibody PHF1 revealed additional intracellular tau inclusions in the molecular layer of the dentate gyrus in PiD and to a lesser extent in PSP while astrocytic plaques were apparent in CBD, all of which were negative for GT-38. Neuronal and astrocytic tau inclusions were present in the pons in cases of CBD and PSP that were similarly negative for GT-38. These results verify the selectivity of GT-38 for AD-tau pathology in the presence of comorbid FTLD-tau pathology.

### GT-38 binding requires 3R and 4R tau isoforms in pathological tau aggregates

To further validate GT-38 selectivity, we performed co-immunofluorescent (co-IFC) staining with GT-38 and 3R- or 4R-tau specific antibodies since AD-tau NFTs are comprised of all six human tau isoforms, including both 3R- and 4R- tau, while PiD aggregates are comprised primarily of 3R-tau and CBD and PSP are comprised of primarily 4R-tau. Previously, co-IFC labeling with isoform specific 3R- and 4R-tau antibodies was hindered by the fact that GT-38, RD3, and RD4 were all mouse IgG1 isotype [18]. Therefore, we generated a mouse IgG2a version of GT-38 containing the identical variable heavy (V_H_) and variable light (V_L_) domains as the original GT-38 hybridoma clone.

**Fig. 2:**
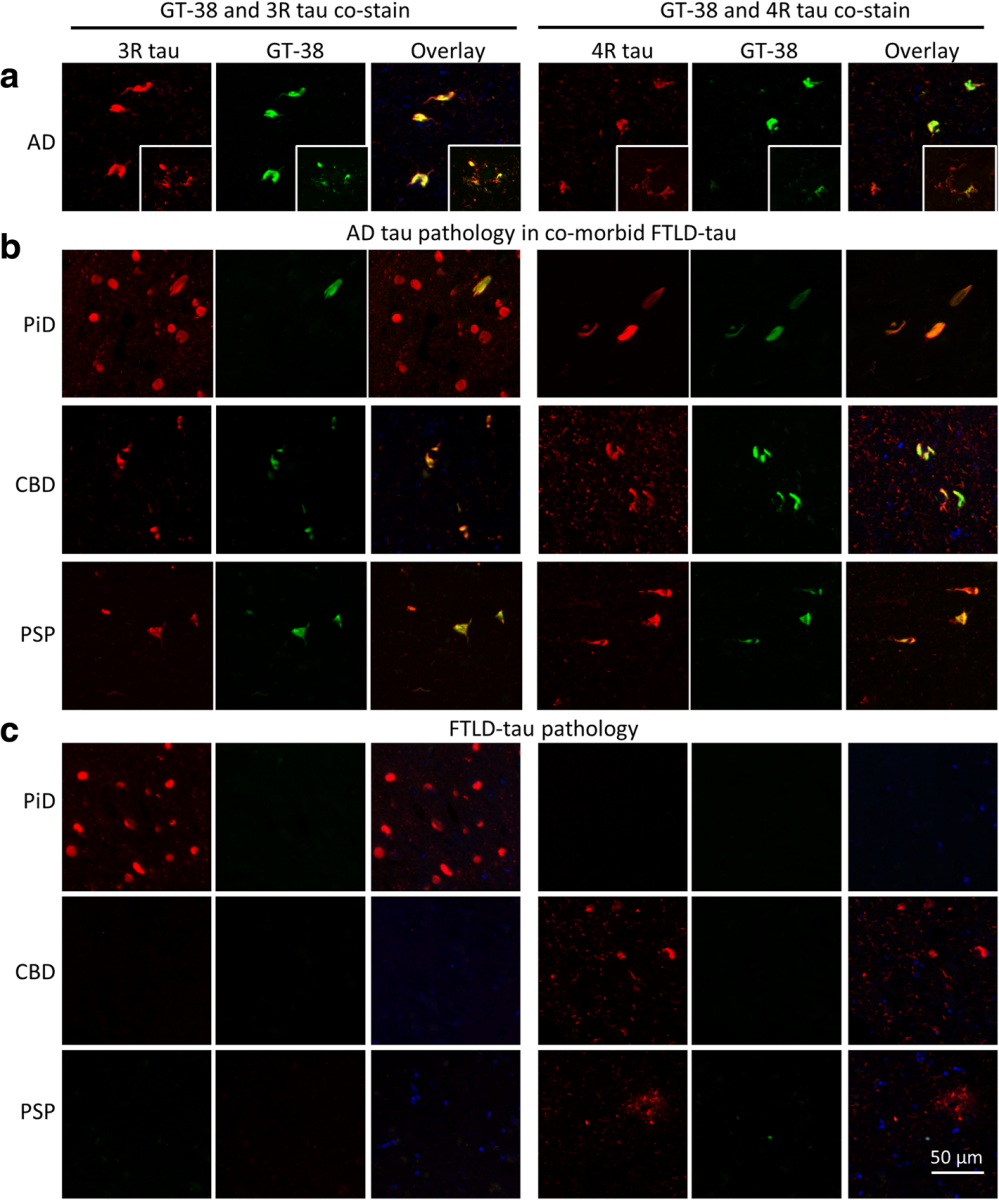
GT-38 detects pathological tau aggregates containing 3R and 4R tau isoforms. Co-immunofluorescent staining of GT-38 with 3R- or 4R-tau specific antibodies in **a** AD midfrontal cortex, **b** CBD, PSP, and PiD hippocampus in regions with co-occurring AD-tau pathology, demonstrating complete colocalization with 4R tau in the context of PiD and complete colocalization with 3R tau pathology in the context of CBD and PSP, **c** GT-38 negative regions in CBD, PSP, and PiD hippocampus without concomitant AD-tau pathology, but with tau pathology comprised of only 3R- or 4R-tau respectively

To directly assess the isoform specificity of GT-38 binding, we performed co-IFC staining of tissue sections from patients with AD, CBD and PSP using the IgG2a version of GT-38 in combination with mouse IgG1 3R- or 4R-tau specific antibodies. In AD tissue, GT-38 highly colocalized with both 3R- and 4R-tau, particularly in dense NFTs (Fig. 2a), while colocalization was also observed in neuropil threads and neuritic plaques. In early-stage regions of AD-tau deposition [7], we often found GT-38 reactivity in the form of NFTs and threads, suggesting co-morbid AD-tau pathology in FTLD-tau. In PiD GT-38 did not detect the 3R-tau positive Pick bodies, but completely colocalized with 4R-tau positive NFT-tau pathology (Fig. 2b). In CBD and PSP, GT-38 did not detect the astrocytic 4R tau pathology, yet stained intraneuronal NFTs in regions positive for 3R-tau pathology that completely co-localize with GT-38 (Fig. 2b). In PiD hippocampal sections with dense 3R-tau positive Pick bodies and no 4R-tau staining, GT-38 was negative and in CBD and PSP hippocampal regions with 4R-tau predominant astrocytic and neuronal pathology without 3R-tau inclusions, GT-38 staining was negative (Fig. 2c). Together, these findings indicate that GT-38 selectively detects pathological tau aggregates comprised of both 3R- and 4R-tau but does not detect pathological FTLD-tau associated aggregates containing only 3R- or 4R-tau alone. Importantly, these studies further validate GT-38 selectively detects AD-tau pathology in the presence of co-morbid FTLD-tau.

### Braak staging AD-tau in comorbid FTLD-tau

**Fig. 3:**
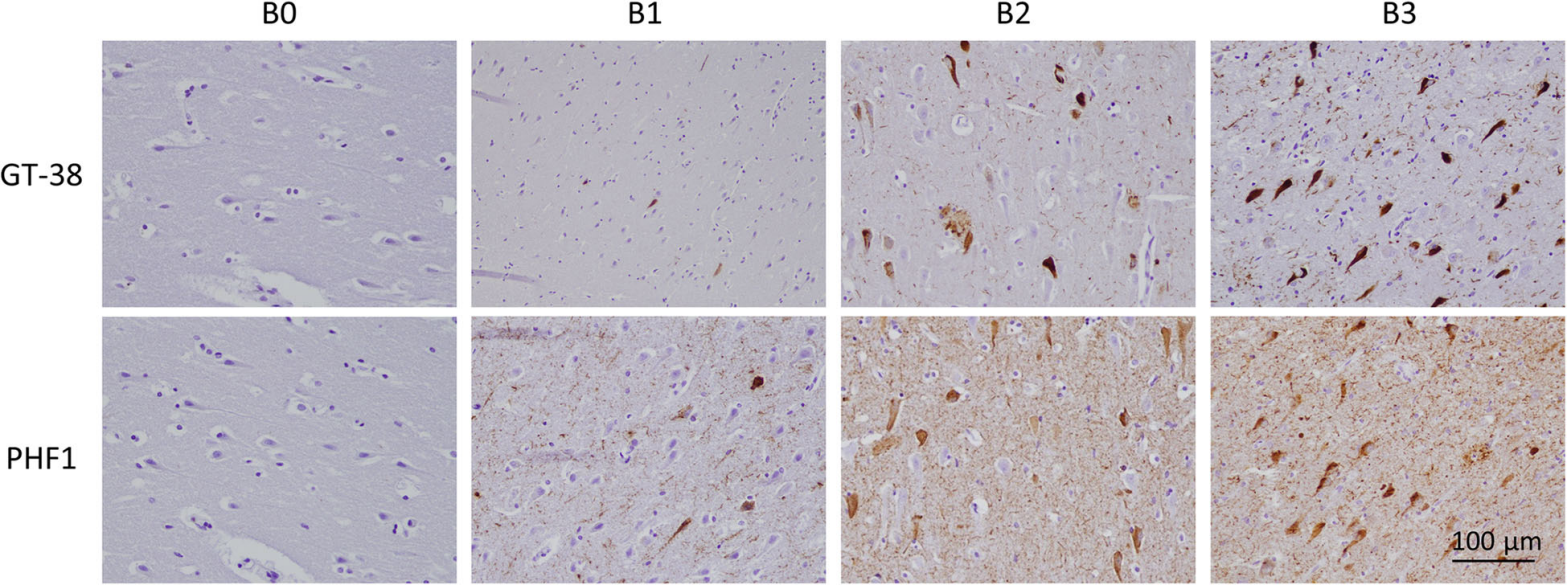
GT-38 validation for Braak staging. GT-38 IHC staining of AD-tau pathology compared to the diagnostic standard antibody PHF1 detects NFTs to a similar extent, but fewer neuropil threads in CA1 regions of hippocampal tissue sections from cases of varying Braak stages (B0-B3) and AD-tau severity

Based on the unique selectivity of GT-38 for AD-tau pathology, our goal was to determine the extent of co-morbid AD in a cohort of 180 people with neuropathologically confirmed FTLD-tau. We first verified that GT-38 detects AD-tau pathology with similar sensitivity to the current benchmark diagnostic antibody, PHF1, which detects tau phosphorylated at Ser396 and Ser404 [21]. We examined the immunoreactivity of GT-38 and PHF1 to pathological tau aggregates in CA1 of the hippocampus from a cohort of patients with AD and no cognitive impairment to assess the concordance of these antibodies in a range of varying Braak stages (Fig. 3). We found similar sensitivity for detection of tau NFTs between GT-38 and PHF1, neuritic plaques were also detected by GT-38, whereas neuropil staining was abundantly stained by PHF1 and to a lesser extent by GT-38. These findings supported the use of GT-38 for Braak staging and demonstrated similar sensitivity as PHF1 for detection of AD-tau.

We next performed GT-38 IHC staining to assigned Braak stages of AD-tau in a cohort of 180 patients with FTLD-tau and tissue available in our brain bank. Demographics of the FTLD-tau patient cohort are shown in (Table 1) including, age at death, sex, disease duration, post mortem interval (PMI), clinical phenotypes, APOE haplotype, CERAD score as measure of Aβ plaque load, and GT-38 defined AD-tau Braak stages determined in this study. Detailed clinical variant subtypes of PSP have been defined in accordance with the recent Movement Disorder Society clinical diagnosis criteria for PSP [25]. Based on GT-38 staining of AD-tau pathology in hippocampus, entorhinal cortex, and visual cortex, Braak staging was performed in accordance with standard diagnostic criteria that previously utilized phospho-tau specific antibodies [6]. GT-38 Braak staging and CERAD scores were evaluated to designate the level of AD neuropathological change (ADNCP) as “no”, “low”, “intermediate”, or “high” based on NIA-AA guidelines for the neuropathological assessment of AD [42].

**Table 1:** Summary of patient demographics and Braak staging of AD-tau

	CBD *N* = 49	PSP *N* = 109	PiD *N* = 22
Sex	M = 19	M = 69	M = 13
	F = 30	F = 40	F = 9
Age at death (years)	67.5 (9.5)	76 (71, 81)^*a*^	67 (61, 74)
Age at onset (years)	61.6 (9.3)	67 (62,74)^*a*^	57.8 (11.1)
Disease duration (years)	6.0 (2.5)	7 (4, 10)^*b*^	9.0 (4.0)
PMI (hours)	11 (5, 17)	12 (6, 19)	12.5 (7, 20)
Brain weight (g)	1139 (139)	1213 (1121, 1304)^*a*^	1026 (161)
Clinical phenotype	CBS = 18	PSP-RS = 61	CBS = 2
	PSP = 12	PSP-SL = 6	PPA = 1
	PPA = 9	PSP-CBS = 8	bvFTD-FTLD = 8
	bvFTD-FTLD = 3	PSP-F = 14	FTD-NOS = 10
	FTD- NOS = 4	PSP-*P* = 8	DLB = 1
	AD = 1	PSP-PGF = 1	
	DLB = 1	Other cognitive = 8	
	Undetermined = 1	Other motor = 3	
APOE haplotype	E2/E2 = 0	E2/E2 = 2	E2/E2 = 1
	E2/E3 = 8	E2/E3 = 16	E2/E3 = 1
	E2/E4 = 0	E2/E4 = 5	E2/E4 = 0
	E3/E3 = 33	E3/E3 = 63	E3/E3 = 16
	E3/E4 = 7	E3/E4 = 12	E3/E4 = 3
	E4/E4 = 1	E4/E4 = 3	E4/E4 = 0
	N/A = 0	N/A = 8	N/A = 1
CERAD	0 = 31	0 = 53	0 = 17
	1 = 9	1 = 15	1 = 1
	2 = 3	2 = 14	2 = 1
	3 = 2	3 = 17	3 = 2
	N/A = 4	N/A = 10	N/A = 1
Thal phase	0 = 27	0 = 37	0 = 14
	1 = 16	1 = 32	1 = 3
	2 = 3	2 = 21	2 = 4
	3 = 3	3 = 16	3 = 1
	N/A = 0	N/A = 3	N/A = 0
Braak stage	B0 = 24	B0 = 28	B0 = 13
	B1 = 21	B1 = 50	B1 = 7
	B2 = 4	B2 = 25	B2 = 1
	B3 = 0	B3 = 6	B3 = 1
AD Neuropathological change	Not = 27	Not = 38	Not = 14
	Low = 19	Low = 50	Low = 7
	Intermediate = 3	Intermediate = 15	Intermediate = 0
	High = 0	High = 4	High = 1

Variables with normal distribution are reported as mean (standard deviation) and variables that are non-normally distributed are reported as median (first quartile, third quartile)

*PMI* post mortem interval from death to autopsy, *CERAD* consortium to establish a registry for Alzheimer’s disease, *CBS* corticobasal syndrome, *PPA* primary progressive aphasia, *bvFTD-FTLD* behavioral variant frontotemporal dementia – frontotemporal lobar degeneration, *DLB* dementia with Lewy bodies, *FTD-NOS* frontotemporal dementia not otherwise specified, *RS* Richardson’s syndrome, *SL* speech and language disorders, *F* frontal lobe cognitive or behavioral presentation, *P* Parkinsonism, *PGF* progressive gait freezing, *N/A* not available

^a^*p* < 0.01 difference between PSP and CBD and PiD

^b^*p* < 0.05 difference between PSP and CBD and PiD

**Fig. 4:**
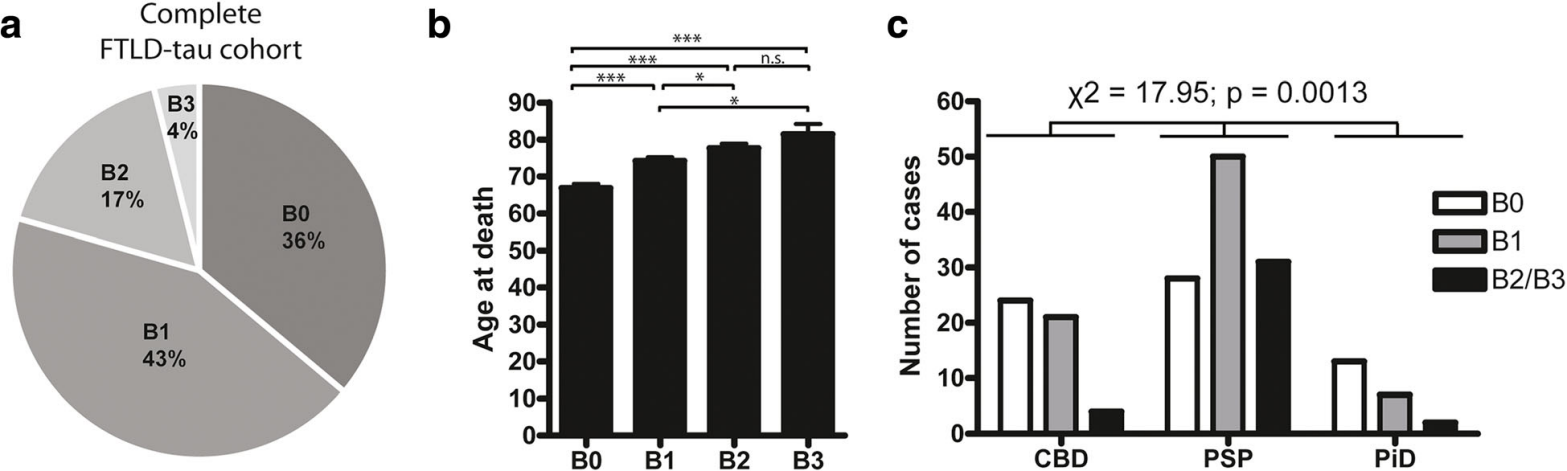
Braak staging of FTLD-tau cohort with GT-38. AD-tau NFT burden was assessed in the hippocampus, pons/locus coeruleus, and visual cortex of 180 FTLD-tau cases and staged Braak 0–3. **a** The relative distribution of Braak stages across the entire cohort. **b** Age at death for each Braak stage (B0 *n* = 65, B1 *n* = 78, B2 *n* = 30, B3 *n* = 7) *** *p* < 0.001; * *p* < 0.05; n.s. = not significant; two-tailed t-test. **c** PSP cases (B0 = 28, B1 = 50, B2/B3 = 31) had significantly higher frequency of AD-tau (χ^2^ (4, *n* = 180) = 17.95; *p* = 0.0013) compared to CBD (B0 = 24, B1 = 21, B2/B3 = 4) and PiD (B0 = 13, B1 = 7, B2/B3 = 2)

FTLD-tau patient groups did not differ in postmortem interval yet there were statistically significant differences in age at onset (*p* < 0.001), disease duration (*p* = 0.012), and age at death (*p* < 0.001) across the three FTLD-tau groups. Planned post-hoc tests between individual groups revealed that PSP had later age at onset and age at death compared to CBD and PiD and no statistically significant differences between CBD and PiD. Brain weight of PiD and CBD cases were reduced in comparison to PSP (*p* < 0.01). Overall, AD-tau pathology was detected in 64% of FTLD-tau cases (43% with B1, 17% with B2 and 4% with B3) (Fig. 4a). Patients with higher Braak stages were significantly older at the time of death (Fig. 4b). To test whether AD-tau co-pathology was more frequent within a specific FTLD-tau subtype, we assessed the distribution of Braak stages in each FTLD-tau subtype. Braak stage B2 and B3 groups were combined due to low frequency and chi squared test was performed. This analysis found increased frequency of high Braak stages in the PSP group compared to CBD and PiD (χ^2^(4, *n*=180) =17.95; *p* = 0.0013) (Fig. 4c).

### AD-tau pathology increases with neuritic plaques

To determine whether increased Braak stages corresponded with amyloid-beta (Aβ) plaque measures of AD pathology, we assessed the relationship between GT-38 assigned AD-tau Braak stages with the INDD records of Aβ plaque scores using the CERAD. We examined the frequency of CERAD scores in our GT-38 defined Braak AD-tau stages and found an increased frequency of patients with high plaque burden (CERAD C2/3) among those patients with Braak AD-tau B2/3 compared to those with low Braak B0/1 (χ^2^(1, *n*=165)=17.76; *p* < 0.001). Univariate logistic regression found high CERAD plaque scores (C2/3) predictive of high AD Braak tau stages (B2/3) (OR = 5.31, 95% CI = 2.34-12.09, *p* < 0.001) (Table 2). These findings provide additional evidence that tau identified by GT-38 corresponds with AD co-pathology in FTLD with increasing Aβ plaque load with increased AD Braak stages detected by GT-38.

**Table 2:** Multivariate regression models to predict post-mortem AD-tau pathology

Univariate Models					
Variable	Odds ratio	95% confidence interval	*P*-value	*R* ^*2*^	BIC
Neuropathological diagnosis	2.77	1.34–5.71	0.006	0.055	183.23
Gender	1.11	0.54–2.30	0.777	< 0.001	193.19
Age at death	1.12	1.06–1.18	< 0.001	0.119	171.50
MAPT haplotype H1/H1	0.89	0.31–2.56	0.836	< 0.001	175.52
APOE4	1.70	0.68–4.24	0.259	0.007	174.35
CERAD score C2/C3^*a*^	5.31	2.34–12.09	< 0.001	0.096	159.55
Final Multivariate Model					
Variable	Odds ratio	95% confidence interval	*P*-value	*R*^*2*^ = 0.164	BIC = 153.38
Age at death	1.09	1.03–1.15	0.002	–	–
CERAD score C2/C3^*a*^	3.75	1.58–8.89	0.003	–	–
Intercept	0.0002	3.2 × 10^−6^, 0.0155	< 0.001	–	–

Table displays univariate associations between negligible-low AD-tau (B0/B1 = 0) and medium-high AD-tau (B2/B3 = 1) and variables in the upper panel and the optimal multivariate model in the lower panel. Based on 165 observations. Model *R*^*2*^ = 0.1639, *p* < 0.0001. *BIC* Bayesian information criteria

^*a*^reference category = CERAD C0/C1

### Multivariate analysis of AD-tau co-pathology in FTLD-tau

To determine the strongest correlates of AD co-pathology in FTLD-tau we used multivariate regression modeling to test the association of neuropathology group (i.e. PSP, CBD, PiD), age at death, sex, CERAD score, APOE and MAPT genotype (Table 2). We found significant independent associations of the neuropathology group (OR=2.77, 95% CI=1.34-5.71), age at death (OR=1.12, 95% CI=1.06-1.18), and CERAD scores, reported above. The optimal model to predict AD co-pathology included age at death (OR=1.09; 95% CI=1.03-1.15; *p*=0.002), and CERAD score (OR=3.75, 95% CI=1.58-8.89;*p*=0.003) (Table 2). Thus, the association between PSP and higher AD co-pathology on univariate analyses appears to be driven by age-related factors.

### CSF and pathological AD-tau

**Fig. 5:**
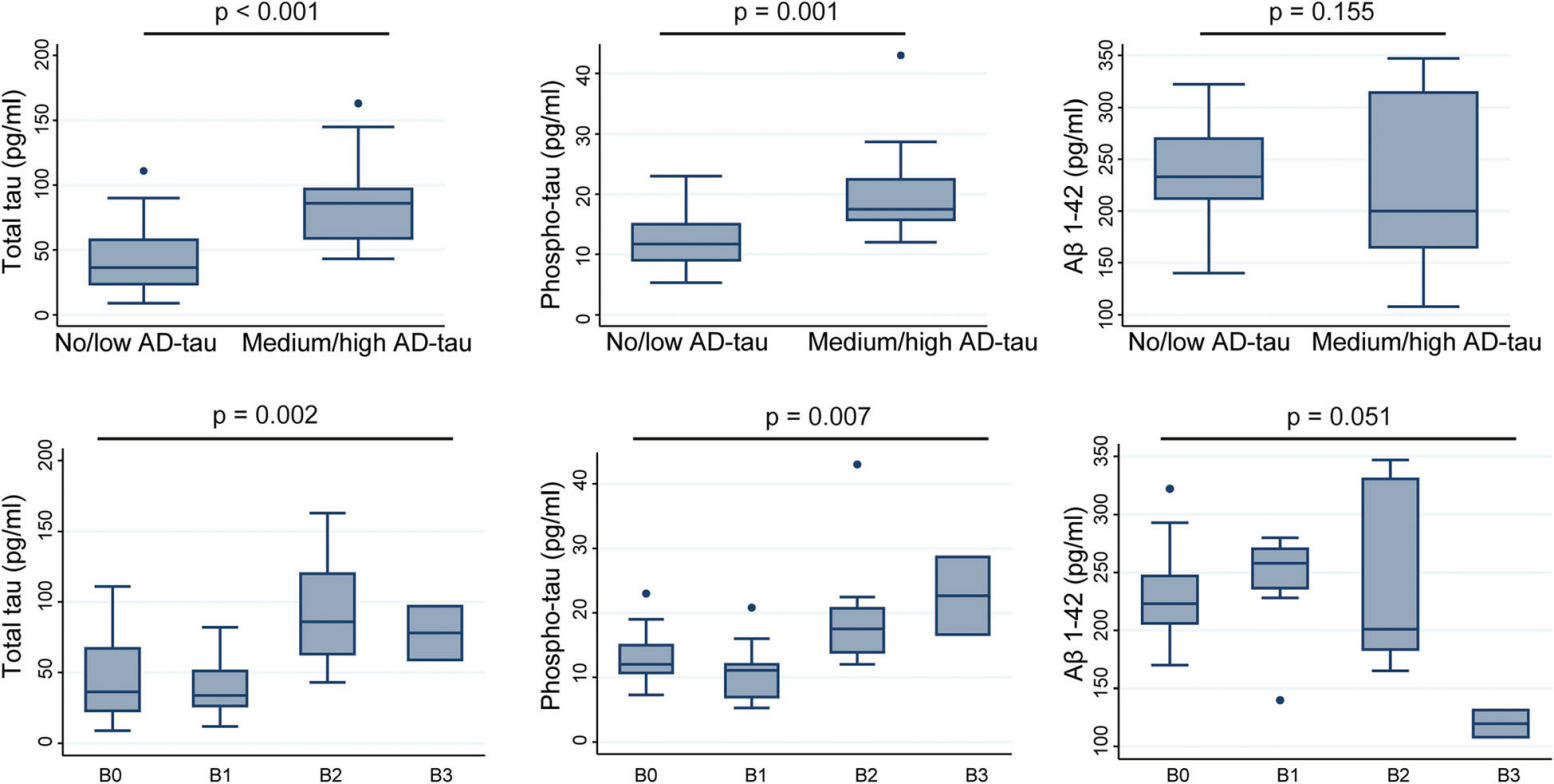
CSF levels of t-tau and p-tau are elevated in medium-high AD-tau Braak stage group defined by GT-38. Box plots of total tau, phosphorylated tau, and Aβ_1–42_ CSF levels for negligible-low AD-tau and medium-high AD-tau groups demonstrate statistically significant elevation of t-tau (*p* < 0.001) and p-tau (*p* = 0.001) in medium-high AD-tau group but a non-statistically significant trends towards decreased Aβ_1–42_ (*p* = 0.155, Mann-Whitney rank sum test)

Antemortem CSF measurement of p-tau, t-tau, and Aβ_1-42_ were available for a subset of the FTLD-tau cases Braak staged with GT-38. We previously reported higher CSF tau levels in FTLD-tau with AD co-pathology detected by Thioflavin-S and amyloid IHC [33, 54]. Here we test the association of AD co-pathology detected by GT-38 in FTLD-tau with antemortem CSF tau measurements for cross-validation of our approach to AD Braak tau staging in FTLD-tau. There was significantly greater levels of t-tau (mean=89.98 pg/ml, SD=36.70 pg/ml) and p-tau (mean=20.45 pg/ml, SD=9.31 pg/ml) in CSF of patients with medium-high AD-tau pathology, than patients with negligible-low AD-tau t-tau (mean=43.04 pg/ml, SD=25.40 pg/ml) and p-tau (mean=11.90 pg/ml, SD=4.48 pg/ml) (*p*≤0.001 both) (Fig. 5). There was a non-statistically significant trend towards decreased Aβ_1-42_ CSF levels between groups (*n*=19 negligible-low AD-tau, n=10 medium-high AD-tau; *p*=0.155 Mann-Whitney Rank Sum test). Lastly, patients with the most severe AD-tau Braak stage B3 had lower Aβ_1-42_ CSF (mean=119.75 pg/ml, SD=16.61) compared to those without AD-tau (mean=232.32, SD=42.38).

### Clinicopathological correlation

In an exploratory clinicopathological analysis, we examined available baseline MMSE scores in our largest FTLD-tau pathology group (PSP) across GT-38 defined AD-tau Braak stages (Braak B0 *n*=6 median/IQR=28.5/27.25-29.25 ; Braak B1 *n*=18, median/IQR=26.0/22.5-27.0; Braak B2/3 *n*=9, median/IQR=27.0/25.5-28.5) and found a significant association of AD Braak stage with lower MMSE score at baseline (H=6.8, df=2, *p*=0.03). Planned post-hoc comparisons between groups found that Braak B0 had a higher baseline MMSE compared to B1 (Z=2.4, *p*=0.02) and to the combined GT-38 AD-tau co-pathology group (i.e. B1-3 n=27, median/IQR=26.0/23.0-28.0 ; Z=2.2, *p*=0.03). We did not find an association of GT-38 AD Braak groups with clinical phenotype (λ^2^=0.9, *p*>0.1) or age at testing (H=4.2, df=2, *p*>0.1).

## Discussion

Here we studied AD co-pathology in a large autopsy cohort of FTLD-tau using a novel mAb specific for AD-tau pathology. We find that AD-tau co-pathology is common in FTLD-tau and is associated with age and Aβ plaque co-pathology. Further, we find converging evidence for specificity for GT-38 to detect postmortem AD-tau through comparison with antemortem CSF tau levels. Finally, we find preliminary data to suggest AD-tau co-pathology in PSP may influence cognitive outcomes. These data add to the growing literature of mixed pathology in age-associated neurodegenerative diseases and have important clinical implications.

These studies provide essential validation of GT-38 as a novel tool to elucidate the currently understudied neuropathological co-occurrence of AD in other tauopathies. While AD-tau pathology can be partially differentiated from FTLD-tau based on regional distribution, cell type specificity, and morphology, this distinction is often challenging using morphological criteria alone as intraneuronal inclusions are also common in CBD, PSP, and PiD [29]. Previous studies have utilized Thio S amyloid binding dye to determine the Braak stage of AD-tau in a small cohort of patients with FTLD-tau and Lewy body disorders [33, 35]. However, Thio S is not specific for tau and binds to Aβ and TDP-43 [3, 38]. Alternatively, 3R- and 4R-tau specific antibodies can be used to determine the isoform composition of tau pathologies but requires staining with multiple antibodies, are fixation condition dependent, and stain non-pathological tau to an extent [13]. A rare subset of FTDP-17 cases containing MAPT mutations contain 3R- and 4R-tau isoform bearing inclusions and based on limited availability, we have not been able to fully characterize the selectivity of GT-38 towards these tau aggregates [16, 43, 48]. Nevertheless, GT-38 provides a robust, single mAb to selectively detect 3R- and 4R-tau pathology for assessment of Braak staging in the presence of co-morbid FTLD-tau.

Co-morbid AD-tau pathology was present in 64% of patients of FTLD-tau examined, albeit mostly low level Braak B1 restricted to regions of the entorhinal cortex and CA1 of the hippocampus. Predictably, age at death was the strongest predictor of AD-tau pathology among the FTLD-tau cohort in univariate logistic regression models, as aging is the greatest risk factor for development of AD [22, 45]. Furthermore, the presence of amyloid plaques was a strong predictor of AD-tau pathology strengthening confidence in GT-38 selective detection of AD relevant pathological tau species since Aβ plaque deposition generally precedes tau aggregates in neocortex of patients with AD [51]. PSP cases more frequently displayed high Braak stage AD-tau compared to CBD or PiD, however this effect was non-significant when accounting for age at death and CERAD score in a multivariate logistic regression model. This finding indicates that the advanced age of the PSP cohort was likely influential for our observations of increased frequency of comorbid AD-tau rather than an underlying biological association of PSP related tauopathy to potentially enhance or initiate AD-related tauopathy. Further work in animal and cell models of tau propagation are needed to test the potential for FTLD-tau strains or pathological conformers to interact with AD related tau pathology. In our optimal multivariate logistic regression model to predict high Braak stage AD-tau pathology we found both age at death and CERAD score as significant predictors, suggesting that both age and amyloid-related mechanisms could contribute to AD related-tauopathy in FTLD-tau. However, of these FTLD-tau cases with significant AD-tau copathology we found 7 of 31 PSP and 1 of 4 CBD cases with Thal amyloid and CERAD plaque scores of 0 or 1, which meet diagnostic criteria for probable or possible PART [9]. The pathological distinction of AD and PART, remains controversial but both are distinct processes from FTLD tauopathy that are important to distinguish in postmortem tissue [12].

Here our detection of AD-tau copathology by GT-38 has demonstrable biomarker alterations during life, as GT-38 identified AD-tau pathology correlated with increased t-tau and p-tau in CSF. Despite the fact that CSF tau fragments may not be directly incorporated into tangle pathology, postmortem neuropathological assessment and *in vivo* positron emission tomography (PET) find AD-tau pathology are related to increased CSF t-tau and p-tau levels [5, 8, 17, 33]. Previously we have shown AD copathology can influence CSF values in FTLD, and these data cross validate the specificity for AD-tau and further emphasize the need for detection of AD copathology *in vivo* [33, 54]. While p-tau, t-tau, and Aβ levels in CSF differentiate AD from healthy controls and FTLD-tau to an extent, future efforts using tau strain specific mAbs to detect AD-tau in CSF has potential to provide great diagnostic value for living patients [2, 10, 50, 52]. Although great progress has been made identifying tau specific PET ligands, there are still challenges of off-target binding and high-inter patient variability, therefore a need exists for additional ligands and GT-38 provides a potential approach to develop AD-tau specific targeted PET ligands [40, 46, 49].

The common co-occurrence of overlapping neuropathologies confounds the clinical diagnoses of various dementia [34]. In our preliminary analysis using rare, autopsy-confirmed, neuropsychological data in a general screening tool for global cognition (MMSE) we found an association of worse performance at baseline with the presence of AD-tau co-pathology in PSP as detected by GT-38. While our PSP group was clinically heterogeneous (Table 1), we did not find an association between cognitive vs motor clinical phenotypes or age in our data set. While this important data represents coordinated effort of capturing harmonized clinical assessments across cognitive and motor subspecialty clinics, we had limited overall MMSE data and lacked sufficient data to test specific cognitive domains in this cohort. Future work with detailed prospective antemortem clinical assessments capturing the broad range of clinical expression of dementia in FTLD-tau (i.e. social cognition, language, spatial functioning, apraxia) followed to autopsy are needed to determine the specific clinical features of dementia associated with AD co-pathology in FTLD-tau. Nevertheless, these initial results suggest AD-tau co-pathology may influence cognitive outcomes in FTLD-tau.

## Conclusion

The data presented here demonstrate the utility of AD-tau specific mAb GT-38 for Braak staging AD pathology in the context of FTLD-tau. GT-38 staining provides a robust and simple tool to neuropathologically differentiate AD specific tau pathology to further elucidate the contribution of AD-tau in comorbid neurodegenerative diseases. In addition, it remains to be determined whether the 3R- and 4R-tau epitope present in AD is recapitulated in other non-age related tauopathies comprised of six tau isoforms such as traumatic brain injury (TBI) or chronic traumatic encephalopathy (CTE). The findings presented here, validate the use of GT-38 in postmortem autopsy tissue and suggest exciting potential for detection of AD-tau in living subjects via CSF or as a PET ligand.

## References

[1] Armstrong MJ, Litvan I, Lang AE, Bak TH, Bhatia KP, Borroni B (2013). Criteria for the diagnosis of corticobasal degeneration. Neurology.

[2] Bian H, Van Swieten JC, Leight S, Massimo L, Wood E, Forman M (2008). CSF biomarkers in frontotemporal lobar degeneration with known pathology. Neurology.

[3] Bigio EH, Wu JY, Deng HX, Bit-Ivan EN, Mao Q, Ganti R (2013). Inclusions in frontotemporal lobar degeneration with TDP-43 proteinopathy (FTLD-TDP) and amyotrophic lateral sclerosis (ALS), but not FTLD with FUS proteinopathy (FTLD-FUS), have properties of amyloid. Acta Neuropathol.

[4] Binder LI, Frankfurter A, Rebhun LI (1985). The distribution of tau in the mammalian central nervous system. J Cell Biol.

[5] Blennow K, Hampel H, Weiner M, Zetterberg H (2010). Cerebrospinal fluid and plasma biomarkers in Alzheimer disease. Nat Rev Neurol.

[6] Braak H, Alafuzoff I, Arzberger T, Kretzschmar H, Del Tredici K (2006). Staging of Alzheimer disease-associated neurofibrillary pathology using paraffin sections and immunocytochemistry. Acta Neuropathol.

[7] Braak H, Braak E (1991). Neuropathological stageing of Alzheimer-related changes. Acta Neuropathol.

[8] Buerger K, Ewers M, Pirttila T, Zinkowski R, Alafuzoff I, Teipel SJ (2006). CSF phosphorylated tau protein correlates with neocortical neurofibrillary pathology in Alzheimer's disease. Brain.

[9] Crary JF, Trojanowski JQ, Schneider JA, Abisambra JF, Abner EL, Alafuzoff I (2014). Primary age-related tauopathy (PART): a common pathology associated with human aging. Acta Neuropathol.

[10] de Souza LC, Lamari F, Belliard S, Jardel C, Houillier C, De Paz R (2011). Cerebrospinal fluid biomarkers in the differential diagnosis of Alzheimer's disease from other cortical dementias. J Neurol Neurosurg Psychiatry.

[11] Drubin DG, Kirschner MW (1986). Tau protein function in living cells. J Cell Biol.

[12] Duyckaerts C, Braak H, Brion JP, Buee L, Del Tredici K, Goedert M (2015). PART is part of Alzheimer disease. Acta Neuropathol.

[13] Espinoza M, de Silva R, Dickson DW, Davies P (2008). Differential incorporation of tau isoforms in Alzheimer's disease. J Alzheimer's Dis : JAD.

[14] Fagan AM, Shaw LM, Xiong C, Vanderstichele H, Mintun MA, Trojanowski JQ (2011). Comparison of analytical platforms for cerebrospinal fluid measures of beta-amyloid 1-42, total tau, and p-tau181 for identifying Alzheimer disease amyloid plaque pathology. Arch Neurol.

[15] Forman MS, Farmer J, Johnson JK, Clark CM, Arnold SE, Coslett HB (2006). Frontotemporal dementia: clinicopathological correlations. Ann Neurol.

[16] Forrest SL, Kril JJ, Stevens CH, Kwok JB, Hallupp M, Kim WS (2017). Retiring the term FTDP-17 as MAPT mutations are genetic forms of sporadic frontotemporal tauopathies. Brain.

[17] Galasko D, Chang L, Motter R, Clark CM, Kaye J, Knopman D (1998). High cerebrospinal fluid tau and low amyloid beta42 levels in the clinical diagnosis of Alzheimer disease and relation to apolipoprotein E genotype. Arch Neurol.

[18] Gibbons GS, Banks RA, Kim B, Changolkar L, Riddle DM, Leight SN (2018). Detection of Alzheimer disease (AD)-specific tau pathology in AD and NonAD Tauopathies by immunohistochemistry with novel conformation-selective tau antibodies. J Neuropathol Exp Neurol.

[19] Goedert M, Eisenberg DS, Crowther RA (2017). Propagation of tau aggregates and neurodegeneration. Annu Rev Neurosci.

[20] Goedert M, Spillantini MG, Potier MC, Ulrich J, Crowther RA (1989). Cloning and sequencing of the cDNA encoding an isoform of microtubule-associated protein tau containing four tandem repeats: differential expression of tau protein mRNAs in human brain. EMBO J.

[21] Greenberg SG, Davies P, Schein JD, Binder LI (1992). Hydrofluoric acid-treated tau PHF proteins display the same biochemical properties as normal tau. J Biol Chem.

[22] Guerreiro R, Bras J (2015). The age factor in Alzheimer's disease. Genome Med.

[23] Guo JL, Narasimhan S, Changolkar L, He Z, Stieber A, Zhang B (2016). Unique pathological tau conformers from Alzheimer's brains transmit tau pathology in nontransgenic mice. J Exp Med.

[24] Gustke N, Trinczek B, Biernat J, Mandelkow EM, Mandelkow E (1994). Domains of tau protein and interactions with microtubules. Biochemistry.

[25] Hoglinger GU, Respondek G, Stamelou M, Kurz C, Josephs KA, Lang AE (2017). Clinical diagnosis of progressive supranuclear palsy: the movement disorder society criteria. Mov Disord.

[26] Hong M, Zhukareva V, Vogelsberg-Ragaglia V, Wszolek Z, Reed L, Miller BI (1998). Mutation-specific functional impairments in distinct tau isoforms of hereditary FTDP-17. Science.

[27] Hutton M, Lendon CL, Rizzu P, Baker M, Froelich S, Houlden H (1998). Association of missense and 5′-splice-site mutations in tau with the inherited dementia FTDP-17. Nature.

[28] Irwin DJ, Brettschneider J, McMillan CT, Cooper F, Olm C, Arnold SE (2016). Deep clinical and neuropathological phenotyping of pick disease. Ann Neurol.

[29] Irwin DJ, Cairns NJ, Grossman M, McMillan CT, Lee EB, Van Deerlin VM (2015). Frontotemporal lobar degeneration: defining phenotypic diversity through personalized medicine. Acta Neuropathol.

[30] Irwin DJ, Cohen TJ, Grossman M, Arnold SE, McCarty-Wood E, Van Deerlin VM (2013). Acetylated tau neuropathology in sporadic and hereditary tauopathies. Am J Pathol.

[31] Irwin DJ, Cohen TJ, Grossman M, Arnold SE, Xie SX, Lee VM (2012). Acetylated tau, a novel pathological signature in Alzheimer's disease and other tauopathies. Brain.

[32] Irwin DJ, Lee VM, Trojanowski JQ (2013). Parkinson's disease dementia: convergence of alpha-synuclein, tau and amyloid-beta pathologies. Nat Rev Neurosci.

[33] Irwin DJ, Lleo A, Xie SX, CT MM, Wolk D, Lee EB et al (2017) Ante mortem CSF tau levels correlate with post mortem tau pathology in FTLD. Ann Neurol 82:247–258 10.1002/ana.24996 PMC5776747 28719018

[34] Irwin DJ, McMillan CT, Toledo JB, Arnold SE, Shaw LM, Wang LS (2012). Comparison of cerebrospinal fluid levels of tau and Abeta 1-42 in Alzheimer disease and frontotemporal degeneration using 2 analytical platforms. Arch Neurol.

[35] Irwin DJ, Xie SX, Coughlin D, Nevler N, Akhtar RS, McMillan CT (2018). CSF tau and beta-amyloid predict cerebral synucleinopathy in autopsied Lewy body disorders. Neurology.

[36] Jicha GA, Bowser R, Kazam IG, Davies P (1997). Alz-50 and MC-1, a new monoclonal antibody raised to paired helical filaments, recognize conformational epitopes on recombinant tau. J Neurosci Res.

[37] Jicha GA, Lane E, Vincent I, Otvos L, Hoffmann R, Davies P (1997). A conformation- and phosphorylation-dependent antibody recognizing the paired helical filaments of Alzheimer's disease. J Neurochem.

[38] Lamy C, Duyckaerts C, Delaere P, Payan C, Fermanian J, Poulain V (1989). Comparison of seven staining methods for senile plaques and neurofibrillary tangles in a prospective series of 15 elderly patients. Neuropathol Appl Neurobiol.

[39] Lee VM, Goedert M, Trojanowski JQ (2001). Neurodegenerative tauopathies. Annu Rev Neurosci.

[40] Lemoine L, Leuzy A, Chiotis K, Rodriguez-Vieitez E, Nordberg A (2018). Tau positron emission tomography imaging in tauopathies: the added hurdle of off-target binding. Alzheimers Dement.

[41] LoPresti P, Szuchet S, Papasozomenos SC, Zinkowski RP, Binder LI (1995). Functional implications for the microtubule-associated protein tau: localization in oligodendrocytes. Proc Natl Acad Sci U S A.

[42] Montine TJ, Phelps CH, Beach TG, Bigio EH, Cairns NJ, Dickson DW (2012). National Institute on Aging-Alzheimer's Association guidelines for the neuropathologic assessment of Alzheimer's disease: a practical approach. Acta Neuropathol.

[43] Murrell JR, Spillantini MG, Zolo P, Guazzelli M, Smith MJ, Hasegawa M (1999). Tau gene mutation G389R causes a tauopathy with abundant pick body-like inclusions and axonal deposits. J Neuropathol Exp Neurol.

[44] Narasimhan S, Guo JL, Changolkar L, Stieber A, McBride JD, Silva LV (2017). Pathological tau strains from human brains recapitulate the diversity of Tauopathies in nontransgenic mouse brain. J Neurosci.

[45] Niccoli T, Partridge L (2012). Ageing as a risk factor for disease. Curr Biol : CB.

[46] Ossenkoppele R, Rabinovici GD, Smith R, Cho H, Scholl M, Strandberg O (2018). Discriminative accuracy of [F-18] flortaucipir positron emission tomography for Alzheimer disease vs other neurodegenerative disorders. Jama-J Am Med Assoc.

[47] Otvos L, Feiner L, Lang E, Szendrei GI, Goedert M, Lee VM (1994). Monoclonal antibody PHF-1 recognizes tau protein phosphorylated at serine residues 396 and 404. J Neurosci Res.

[48] Rosso SM, van Herpen E, Deelen W, Kamphorst W, Severijnen LA, Willemsen R (2002). A novel tau mutation, S320F, causes a tauopathy with inclusions similar to those in Pick's disease. Ann Neurol.

[49] Saint-Aubert L, Lemoine L, Chiotis K, Leuzy A, Rodriguez-Vieitez E, Nordberg A (2017). Tau PET imaging: present and future directions. Mol Neurodegener.

[50] Schoonenboom NS, Reesink FE, Verwey NA, Kester MI, Teunissen CE, van de Ven PM (2012). Cerebrospinal fluid markers for differential dementia diagnosis in a large memory clinic cohort. Neurology.

[51] Selkoe DJ, Hardy J (2016). The amyloid hypothesis of Alzheimer's disease at 25 years. EMBO Molecular Med.

[52] Shaw LM, Vanderstichele H, Knapik-Czajka M, Clark CM, Aisen PS, Petersen RC (2009). Cerebrospinal fluid biomarker signature in Alzheimer's disease neuroimaging initiative subjects. Ann Neurol.

[53] Taniguchi-Watanabe S, Arai T, Kametani F, Nonaka T, Masuda-Suzukake M, Tarutani A (2016). Biochemical classification of tauopathies by immunoblot, protein sequence and mass spectrometric analyses of sarkosyl-insoluble and trypsin-resistant tau. Acta Neuropathol.

[54] Toledo JB, Brettschneider J, Grossman M, Arnold SE, Hu WT, Xie SX (2012). CSF biomarkers cutoffs: the importance of coincident neuropathological diseases. Acta Neuropathol.

[55] Trojanowski JQ, Dickson D (2001). Update on the neuropathological diagnosis of frontotemporal dementias. J Neuropathol Exp Neurol.

[56] Trojanowski JQ, Schuck T, Schmidt ML, Lee VM (1989). Distribution of tau proteins in the normal human central and peripheral nervous system. J Histochem Cytoche.

[57] von Bergen M, Barghorn S, Biernat J, Mandelkow EM, Mandelkow E (2005). Tau aggregation is driven by a transition from random coil to beta sheet structure. Biochim Biophys Acta.

[58] Wang LS, Leung YY, Chang SK, Leight S, Knapik-Czajka M, Baek Y (2012). Comparison of xMAP and ELISA assays for detecting cerebrospinal fluid biomarkers of Alzheimer's disease. J Alzheimer's Dis : JAD.

[59] Xie SX, Baek Y, Grossman M, Arnold SE, Karlawish J, Siderowf A (2011). Building an integrated neurodegenerative disease database at an academic health center. Alzheimer's Dement.

